# Data‐independent acquisition proteomics of cerebrospinal fluid implicates endoplasmic reticulum and inflammatory mechanisms in amyotrophic lateral sclerosis

**DOI:** 10.1111/jnc.16030

**Published:** 2023-12-12

**Authors:** Elizabeth R. Dellar, Iolanda Vendrell, Kevin Talbot, Benedikt M. Kessler, Roman Fischer, Martin R. Turner, Alexander G. Thompson

**Affiliations:** ^1^ Nuffield Department of Clinical Neurosciences University of Oxford Oxford UK; ^2^ Centre for Medicines Discovery, Nuffield Department of Medicine, Target Discovery Institute University of Oxford Oxford UK; ^3^ Nuffield Department of Medicine, Chinese Academy of Medical Sciences Oxford Institute University of Oxford Oxford UK; ^4^ Kavli Institute for Nanoscience Discovery University of Oxford Oxford UK

**Keywords:** amyotrophic lateral sclerosis, biomarker, CSF, DIA, mass spectrometry, proteomics

## Abstract

While unbiased proteomics of human cerebrospinal fluid (CSF) has been used successfully to identify biomarkers of amyotrophic lateral sclerosis (ALS), high‐abundance proteins mask the presence of lower abundance proteins that may have diagnostic and prognostic value. However, developments in mass spectrometry (MS) proteomic data acquisition methods offer improved protein depth. In this study, MS with library‐free data‐independent acquisition (DIA) was used to compare the CSF proteome of people with ALS (*n* = 40), healthy (*n* = 15) and disease (*n* = 8) controls. Quantified protein groups were subsequently correlated with clinical variables. Univariate analysis identified 7 proteins, all significantly upregulated in ALS versus healthy controls, and 9 with altered abundance in ALS versus disease controls (FDR < 0.1). Elevated chitotriosidase‐1 (CHIT1) was common to both comparisons and was proportional to ALS disability progression rate (Pearson *r* = 0.41, FDR‐adjusted *p* = 0.035) but not overall survival. Ubiquitin carboxyl‐terminal hydrolase isozyme L1 (UCHL1; upregulated in ALS versus healthy controls) was proportional to disability progression rate (Pearson *r* = 0.53, FDR‐adjusted *p* = 0.003) and survival (Kaplan Meier log‐rank *p* = 0.013) but not independently in multivariate proportional hazards models. Weighted correlation network analysis was used to identify functionally relevant modules of proteins. One module, enriched for inflammatory functions, was associated with age at symptom onset (Pearson *r* = 0.58, FDR‐adjusted *p* = 0.005) and survival (Hazard Ratio = 1.78, FDR = 0.065), and a second module, enriched for endoplasmic reticulum proteins, was negatively correlated with disability progression rate (*r* = −0.42, FDR‐adjusted *p* = 0.109). DIA acquisition methodology therefore strengthened the biomarker candidacy of CHIT1 and UCHL1 in ALS, while additionally highlighted inflammatory and endoplasmic reticulum proteins as novel sources of prognostic biomarkers.

## Graphical abstract

Dellar et al. explored the utility of library‐free data‐independent acquisition mass spectrometry for proteomic profiling of cerebrospinal fluid in amyotrophic lateral sclerosis, achieving high proteomic depth with limited missing data. This study strengthens Chitotriosidase 1 and Ubiquitin C‐terminal hydrolase L1 as biomarker candidates relating to the aggressiveness of amyotrophic lateral sclerosis, and highlights inflammatory and endoplasmic reticulum proteins as potential novel sources of prognostic biomarkers.

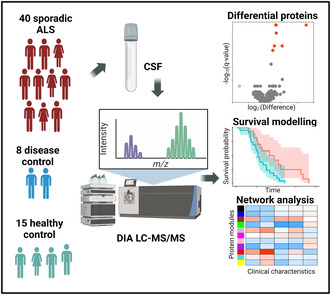

**Abbreviations**
ALS: Amyotrophic Lateral Sclerosis
CSF: Cerebrospinal fluid
DDA: Data‐dependent acquisition
DIA: Data‐independent acquisition
HC: Healthy controls
LC–MS/MS: Liquid chromatography‐tandem mass spectrometry
pNFH: Phosphorylated neurofilament heavy chain

## 1 INTRODUCTION

Amyotrophic lateral sclerosis (ALS) is a fatal neurodegenerative disease in which the death of upper and lower motor neurons results in progressive weakness, typically resulting in death within 3 years after first symptom onset (Westeneng et al., 2018). However, ALS shows marked clinical heterogeneity in relation to its onset, progression and survival (Westeneng et al., 2018), for which biomarkers are a priority area of research. Neurofilaments are cytoskeletal proteins released into the cerebrospinal fluid (CSF) and blood as a result of axonal injury. Levels are elevated in response to a range of neurological pathology (Gaetani et al., 2019; Rosengren et al., 1996), including ALS (Boylan et al., 2013; Oeckl et al., 2020; Rosengren et al., 1996), where they are the leading prognostic biomarker, showing correlation with both disability progression rate and overall survival, and with growing importance in therapeutic trials as an early indicator clinical benefit (Feneberg et al., 2018; Miller et al., 2022; Steinacker et al., 2016; Thompson, Gray, et al., 2022). Given they are non‐specifically elevated following neuronal injury, the diagnostic utility of neurofilament levels is limited, and they provide no information on the upstream causes of neuronal damage in ALS that would enable targeted treatments (Davies et al., 2023).

Although aggregated trans‐active response DNA binding protein 43 kDa (TDP‐43) is the neuropathological hallmark in over 95% of ALS cases, a wide range of upstream pathways have been implicated in mediating neuronal death, including oxidative stress, excitotoxicity, mitochondrial dysfunction, impaired protein homeostasis, endoplasmic reticulum stress, dysregulated RNA transport and metabolism and DNA damage repair (Mead et al., 2022; Neumann et al., 2006). Accumulating evidence also indicates that other cell types in the central nervous system (CNS), including astrocytes, microglia and oligodendrocytes, contribute non‐cell autonomous effects on motor neurons in both a neurotoxic and neuroprotective manner, depending on disease stage (Vahsen et al., 2021). There is a need to establish a range of biomarkers that reflect the complexity of cellular dysfunction associated with ALS pathogenesis. For example, raised levels of glial fibrillary acidic protein (GFAP) in CSF and plasma have been suggested to be indicative of astroglial involvement, and linked to cognitive impairment in ALS as well as in frontotemporal dementia (FTD) and Alzheimer's (Falzone et al., 2022; Zhu et al., 2021). As well as having diagnostic and prognostic value, they may serve as markers of target engagement of potential disease‐modifying therapies as well as indicators of clinical benefit.

Whilst blood is a more accessible source of biomarkers and is ideal to enable monitoring of disease progression, CSF is the closest fluid to the site of dysfunction, so is more suited to biomarker discovery studies (Thompson et al., 2021). As an unbiased approach, shotgun proteomics using data‐dependent acquisition (DDA) liquid chromatography–tandem mass spectrometry (LC–MS/MS) has proved itself a powerful tool for biomarker discovery in ALS, identifying an increase in a set of glial proteins—the chitinase proteins chitotriosidase‐1 (CHIT1), chitinase‐3‐like protein‐1 (CHI3L1 or YKL‐40) and chitinase‐3‐like protein‐2 (CHI3L2 or YKL‐39) (Thompson et al., 2018; Varghese et al., 2013). Additional proteins such as glycoprotein nonmetastatic melanoma protein B (GPNMB), macrophage‐capping protein (CAPG), alpha 1‐antichymotrypsin (SERPINA3) identified in mass spectrometry, and chemokines or cytokines including monocyte chemotactic protein 1 (MCP‐1), macrophage inflammatory protein‐1α and β (MIP‐1α and MIP‐1β), interleukin‐8, and granulocyte colony‐stimulating factor (GM‐CSF) point towards a strong inflammatory involvement in the disease (Baron et al., 2005; Huang et al., 2020; Kuhle et al., 2009; Mitchell et al., 2009; Oeckl et al., 2020; Oh et al., 2023; Yang et al., 2016). However, the temporal profile of inflammatory involvement in ALS is poorly understood, with mixed data on the association of these proteins with survival and disability progression.

As a result of the stochastic nature of DDA LC–MS/MS, more abundant proteins are more readily detected. In CSF, the abundant plasma proteins, such as albumin and immunoglobulins, mask the detection of CNS‐derived proteins, which have much lower absolute abundance (Feneberg et al., 2014). Improving proteomic depth by immuno‐depletion of abundant proteins and strategies combining isobaric labelling with pre‐fractionation have been undertaken, yielding several promising candidate biomarkers including Ubiquitin carboxyl‐terminal hydrolase isozyme L1 (UCHL1) and Neuronal pentraxin‐2 (NPTX2; Collins et al., 2015; Oeckl et al., 2020; Oh et al., 2023) in CSF in ALS. These methods are vulnerable to higher preanalytical variability and a greater proportion of missing values than a typical label‐free workflow, with a subsequent impact on statistical power (Carlyle et al., 2018). More recently, data‐independent acquisition (DIA) methods have been developed, in which multiple precursor ions are fragmented together, thereby reducing bias towards higher abundance proteins, reducing variability, and increasing depth in label‐free analysis (Krasny & Huang, 2021). This approach is more suited to the detection of smaller changes and further allows the assessment of changes at the network level, which better represents the cellular perturbations occurring in the disease than do single proteins.

Here we test the DIA methodology for deep proteomic profiling of CSF samples from a cohort of sporadic ALS patients, compared with healthy and disease‐control groups. We demonstrate that both CHIT1 and UCHL1 abundance are significantly increased and show strong associations with prognosis. Furthermore, we identify the increased abundance of multiple markers of non‐neuronal inflammatory involvement and demonstrate the use of network‐based analysis to distinguish signatures of different aspects of cellular dysfunction involved in pathogenesis.

## 2 METHODS

### 2.1 Participants and sampling

People attending the Oxford Motor Nerve Clinic were diagnosed with ALS according to standard criteria by experienced neurologists (M.R.T., K.T.). Healthy control (HC) participants were typically spouses and friends of those with ALS, while relevant disease controls (DC) were patients seen in Oxford Motor Nerve Clinic with other motor disorders. Demographic and clinical characteristics are listed in Table 1, and diagnoses of disease controls are listed in Table S1. Healthy control participants were excluded if they had a family history of ALS. Exclusion criteria for all participants included age <18 years and contraindication to lumbar puncture, including inherited bleeding disorders, chronic liver disease and use of anticoagulant or antiplatelet drugs and systemic illness at the time of CSF sampling. The samples used in this study received ethical approval from either South Central Oxford Ethics Committee B (08/H0605/85) or NRES Central Committee South Central–Berkshire (14/SC/0083 and 10/H0505/71). All participants provided written consent (or gave permission for a carer to sign on their behalf). CSF samples and clinical information were obtained at the same visit. The disability progression rate was based on the revised ALS Functional Rating Scale (ALSFRS‐R) calculated as (48‐[ALSFRS‐R])/[months from first weakness]. CSF samples were obtained by lumbar puncture following standard clinical procedures. CSF was centrifuged at 2300 *g* for 10 min at 4°C within 2 h of sampling and stored at −80°C until sample preparation.

**TABLE 1:** Participant demographics.

Characteristic	ALS (*n* = 40)	Healthy controls (*n* = 15)	Disease controls (*n* = 8)
Age at sampling (Mean ± SD) (years)	61.8 ± 11.0	56.3 ± 10.3	62.7 ± 15.9
Age at onset (Mean ± SD) (years)	59.9 ± 12.2	–	52.1 ± 26.6
Disease duration (onset to sampling) (Mean ± SD) (months)	22.5 ± 16.4	–	–
Sex (% male)	28 (70%)	8 (53%)	8 (100%)
Onset site (% bulbar)	12 (30%)	–	–
Median disability progression rate (Q1, Q3)	0.54 (0.29–0.97)	–	–

### 2.2 Sample preparation for proteomics

Samples of CSF were thawed on ice and a pooled sample for technical validation was created by combining equal volumes from each individual sample. Samples were digested using Trypsin SMART‐digest plate‐based kit (Thermo Fisher Scientific) according to the manufacturer's protocol. Fifty microlitres of CSF was mixed with 150 μL of SMART digest buffer and added to SMART digest plates containing bead‐immobilised trypsin, and incubated at 70°C on an orbital shaker at 1400 rpm for 2 h. Desalting of digested samples was carried out using SOLAμ SPE plates with 0.1% trifluoroacetic acid and 70% acetonitrile and dried. Samples were resuspended in 60ul 2% acetonitrile/0.1% formic acid prior to analysis.

### 2.3 Mass spectrometry

Peptides were analysed by liquid chromatography–tandem mass spectrometry (LC–MS/MS) using an Ultimate 3000 UHPLC coupled to an Orbitrap Fusion Lumos Mass Spectrometer (both from ThermoFisher). 1.6% of the tryptic peptides were separated on an EASY‐spray PepMap column (2 μm, 75 μm × 50 cm; ThermoScientific) using a 60‐min linear gradient from 2% to 35% buffer B (5% DMSO, 100% acetonitrile, 0.1% formic acid) with a 250 nL/min flow and analysed on the Orbitrap Fusion Lumos. Data were acquired in data‐independent acquisition mode as described previously (Muntel et al., 2019; O'Brien et al., 2023). In brief, MS1 scans were acquired in the Orbitrap with 120k resolution (*m*/*z* 350–1650), an AGC target of 5e5 and a maximum injection time of 20 ms. The full MS events were followed by 40 DIA scan windows per cycle (with variable isolation width) covering the *m*/*z* range from 350 to 1650 (Table S2). The MS/MS scans were acquired in the orbitrap at 30k resolution and normalised HCD set up at 30%. Raw mass spectrometry files were analysed in DIA‐NN v8 using the library‐free approach (Demichev et al., 2019). Spectra were searched against the UniprotSwissprot reviewed human proteome (downloaded February 2021 containing 20 386 sequence), with match‐between runs, 1% peptide false discovery rate and allowing for 1 missed cleavage. N‐terminal excision and oxidation of methionine were included as variable modifications.

### 2.4 Statistical analysis

Data were filtered to exclude protein groups that were present in <50% samples of any one type. Abundance values were log_2_ transformed, normalised using the median abundance of the 90% protein groups with the lowest variance and scaled by median absolute deviation in Python. Data was then imputed by multivariate feature imputation using an iterative method with Sci‐Kit Learn IterativeImputer (Pedregosa et al., 2011). Univariate analysis was carried out using Perseus (2.0.3.0) with Student's *t*‐test with Benjamini‐Hochberg method for false‐discovery rate correction, with a cut‐off of FDR‐adjusted *p* < 0.1 (Tyanova et al., 2016). Volcano plots were produced in Python with the bioinfokit Python package (v2.0.8) (Bedre, 2022).

### 2.5 Weighted correlation network analysis

Weighted correlation network analysis was carried out on imputed data in R (4.2.0) with the weighted gene correlation network analysis (WGCNA) package (Langfelder & Horvath, 2008). A signed network with soft power 4, min module size of 10 and module similarity cut‐height of 0.25 was generated. Intramodular hub proteins were defined as the single proteins with the highest connectivity within each module. Module‐trait associations were generated from pairwise Pearson correlation coefficients, or univariate hazard ratios from Cox hazards models, with Benjamini‐Hochberg method for false‐discovery rate adjustment.

### 2.6 Survival analysis

Survival curves were plotted in R using Survminer package using scaled data, stratifying protein abundance into low and high categories based on mean abundance. Cox proportional hazards modelling was carried out in R using the Survival package (Kassambara et al., 2021).

### 2.7 Gene ontology term enrichment

Gene ontology overrepresentation analysis was carried in R using Webgestalt (Liao et al., 2019). The background reference was set to all detected protein groups within the filtered and deduplicated dataset, giving an effective domain size of 1464. GO:CC, GO:BP and GO:MF were included in the analysis and Benjamini‐Hochberg method for false‐discovery rate was applied, with a cut‐off of 0.05.

### 2.8 ELISA

Phosphorylated neurofilament heavy chain (pNFH) in neat CSF was measured in duplicate with a CE‐labelled sandwich ELISA with a lower detection limit of 270 pg/mL (Euroimmun AG, Lübeck, Germany). The mean intra‐assay CV was 10.26% and interassay CV was 10.13%.

## 3 RESULTS

### 3.1 DIA LC–MS/MS permitted high‐depth robust quantitation of CSF proteins

To validate the DIA approach for biomarker discovery in ALS, we compared CSF samples from sporadic ALS patients (40) with age‐matched healthy controls (15) and relevant disease controls (8) (Table 1; Table S1). Using a previously tested workflow for sample preparation (Thompson et al., 2018) and library‐free DIA acquisition with match‐between‐runs algorithm, 1832 protein groups were quantitated from only 50 μL of CSF, with a mean of 1485 per sample. A total of 873 proteins (48%) were identified in all 63 samples and 1208 (66%) in at least 90% of samples. For group comparisons, a strict filtering was applied, with included proteins quantified in at least 50% of samples in a single group, amounting to 1593 proteins, or 87% of the total. Comparing this list to the Human Protein Atlas showed that 348 proteins (22%) overlapped with the 2685 proteins that are defined as having elevated expression in the brain, 3 of which are classed as having brain‐specific expression (DRAXIN, TMEM132D, IL1RAPL1).

### 3.2 Univariate analysis identified 14 significantly differentially abundant proteins in ALS

**FIGURE 1:**
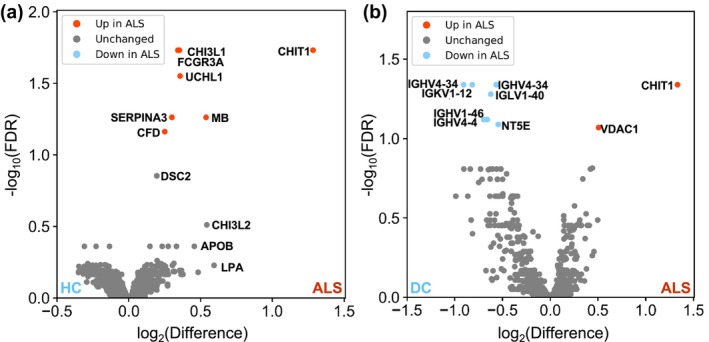
Fourteen differentially abundant proteins in ALS CSF samples. (a, b) Volcano plots showing Log_2_ difference versus FDR‐adjusted *p*‐values for 1593 protein groups quantified using DIA LC–MS/MS with label‐free quantification. Red points indicate protein groups upregulated in ALS versus healthy controls or disease controls, blue points represent downregulated protein groups, at FDR‐adjusted *p* < 0.1. ALS, amyotrophic lateral sclerosis; DC, disease control; FDR, false discovery rate; HC, healthy control.

We then compared protein abundance in sporadic ALS samples to either healthy control or disease control conditions. Univariate analysis identified seven protein groups as significantly different in ALS versus healthy controls and nine in ALS versus disease controls at an FDR threshold of 0.1 (Figure 1a,b). All differentially abundant proteins in ALS versus healthy controls were proteins that were increased in ALS and included CHIT1 and CHI3L1, along with UCHL1 and Alpha 1‐antichymotrypsin (SERPINA3) which have recently been independently identified as significantly higher in ALS patients (Oeckl et al., 2020; Oh et al., 2023). The remaining three protein groups were low‐affinity immunoglobulin gamma Fc region receptor III‐A (FCGR3A), myoglobin (MB) and complement factor D (CFD). CHIT1 was also one of two proteins increased in ALS versus disease controls, alongside the mitochondrial Voltage‐dependent anion‐selective channel protein‐1 (VDAC1). Significantly decreased protein groups corresponded to immunoglobulin proteins (IGHV4‐4, IGKV1‐12, IGHV4‐34, IGLV1‐40, IGHV1‐46), with the exception of 5′‐nucleotidase (NT5E). Several other proteins also showed a sub‐threshold change in abundance, including Apolipoprotein B (APOB) and chitinase‐like protein 2 (CHI3L2) which have previously been linked to ALS in other studies (Thompson et al., 2019; Thompson, Talbot, et al., 2022).

### 3.3 UCHL1 showed strong association with disability progression and survival time

**FIGURE 2:**
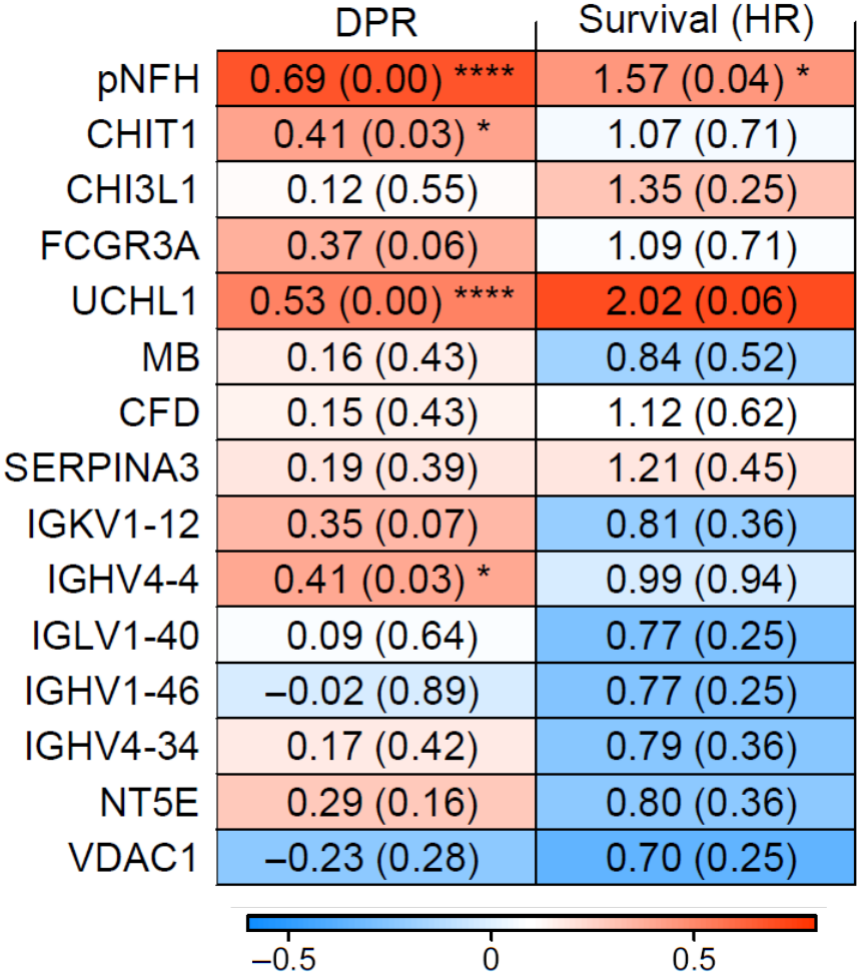
Association of differentially abundant proteins with disability progression rate and survival for ALS patients. Pearson correlation of differentially abundant proteins with disability progression rate (DPR) and univariate hazard ratios (HR), compared to phosphorylated neurofilament heavy (pNFH). UCHL1, Ubiquitin carboxyl‐terminal hydrolase isozyme L1; CHIT1, chitotriosidase; IGHV4‐4, Immunoglobulin heavy variable 4–4; FCGR3A, low‐affinity immunoglobulin gamma Fc region receptor III‐A; IGKV1‐12, Immunoglobulin kappa variable 1–12; NT5E, 5′‐nucleotidase; VDAC1, Voltage‐dependent anion‐selective channel protein 1; SERPINA3, Alpha‐1‐antichymotrypsin; IGHV4‐39, Immunoglobulin heavy variable 4–39; MB, Myoglobin; CFD, Complement factor D; CHI3L1, chitinase‐3‐like protein 1; IGLV1‐40, Immunoglobulin lambda variable 1–40; IGHV1‐46, Immunoglobulin heavy variable 1–46. *FDR < 0.05; ****FDR < 0.0001.

**FIGURE 3:**
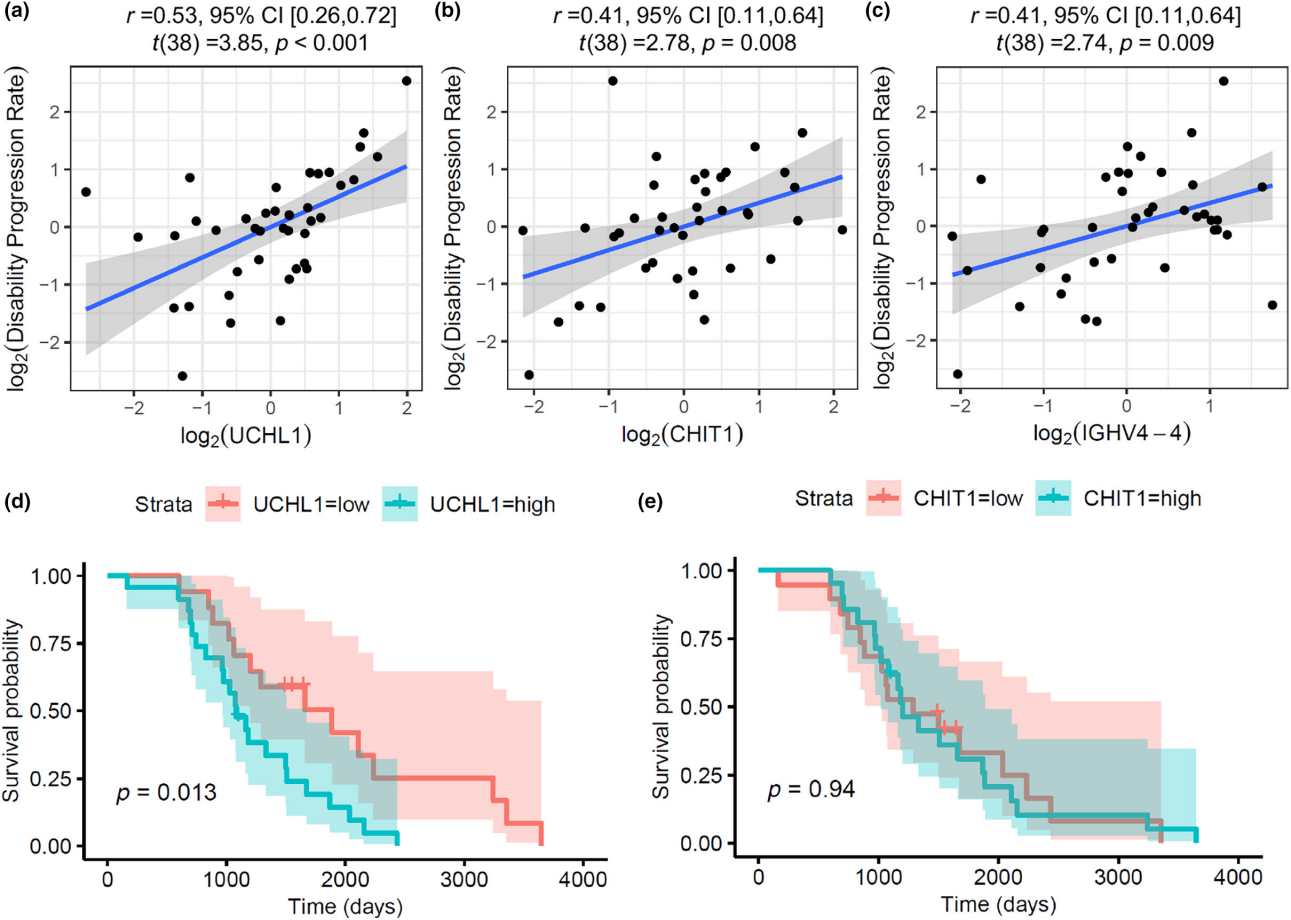
UCHL1 shows a strong association with both disability progression rate and survival. (a–c) Visualisation of linear correlation between CHIT1, UCHL1, and IGHV4‐4 and disability progression rate measured by the rate of decline in revised ALS Functional Rating Scale (ALSFRS‐R). (d, e) Kaplan–Meier survival analysis in ALS, categorised by above or below mean protein abundance of UCHL1 or CHIT1. p‐values for the log‐rank test are shown. CHIT1, chitotriosidase; IGHV4‐4, Immunoglobulin heavy variable 4–4; UCHL1, Ubiquitin carboxyl‐terminal hydrolase isozyme L1.

To further evaluate the use of these 14 differentially abundant proteins as potential biomarkers, we then considered their abundance in relation to disability progression rate, and performed univariate Cox regression modelling on survival, comparing these to ELISA data for phosphorylated neurofilament heavy chain (pNFH; Figure 2). UCHL1, CHIT1 and IGHV4‐4 all showed a strong positive association with disability progression rate (Figures 2 and 3a–c), but all were weaker than the relationship with pNFH, and no relationship was seen with CHI3L1. For survival, pNFH Hazard ratio = 1.57 (FDR = 0.043), and for UCHL1 Hazard ratio = 2.02 (FDR = 0.056) (Figure 2). Using Kaplan–Meier analysis, survival stratified by mean protein abundance was shorter in those with higher UCHL1 (log‐rank *p* = 0.013), but no association was observed with CHIT1 levels (log‐rank *p* = 0.94) (Figure 3d,e).

**FIGURE 4:**
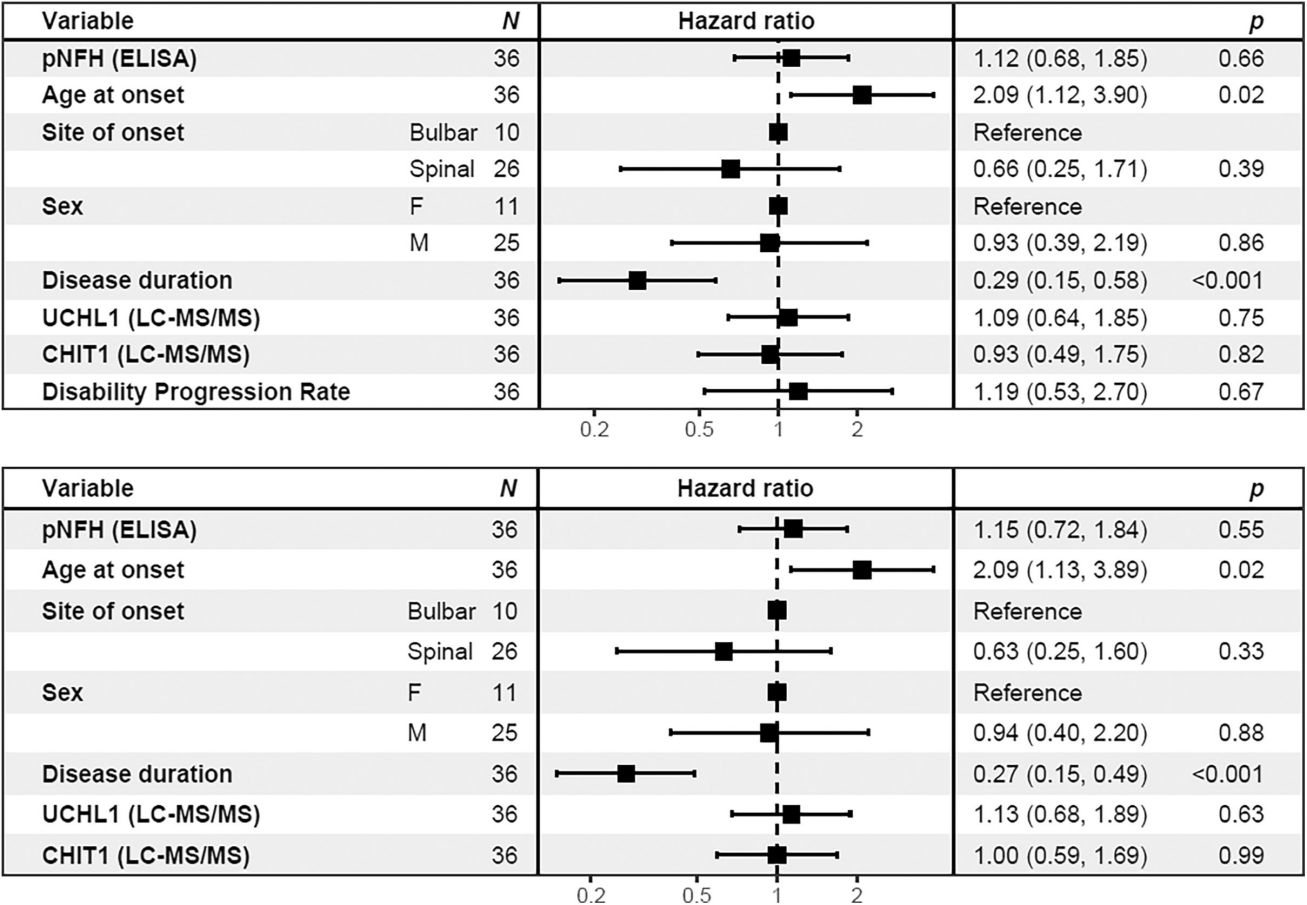
No independent effect of UCHL1 and CHIT1 in Cox Proportional Hazards models. ALS patients at the first visit, with or without the inclusion of disability progression rate as a variable. Neither UCHL1 nor CHIT1 show significant effects in each model. Hazard ratios represent a rise per one standard deviation. CHIT1, chitotriosidase; CI, confidence interval; HR, Hazard ratio; pNFH, phosphorylated neurofilament heavy; UCHL1, Ubiquitin carboxyl‐terminal hydrolase isozyme L1.

Cox proportional hazards models were constructed incorporating available first‐visit clinical characteristics known to be associated with survival in ALS (disease duration at sampling, age at onset, bulbar versus spinal symptom onset, and sex), along with pNFH and LC–MS/MS UCHL1 and CHIT1, with and without disability progression rate as an additional variable (Figure 4). In both models, neither UCHL1 nor CHIT1 showed an independent association with survival. However, UCHL1 was strongly associated with pNFH (*r* = 0.49, *p* = 0.002; Figure S1), further supporting its value as a prognostic marker.

### 3.4 Network analysis identified inflammatory and endoplasmic reticulum involvement in ALS

**FIGURE 5:**
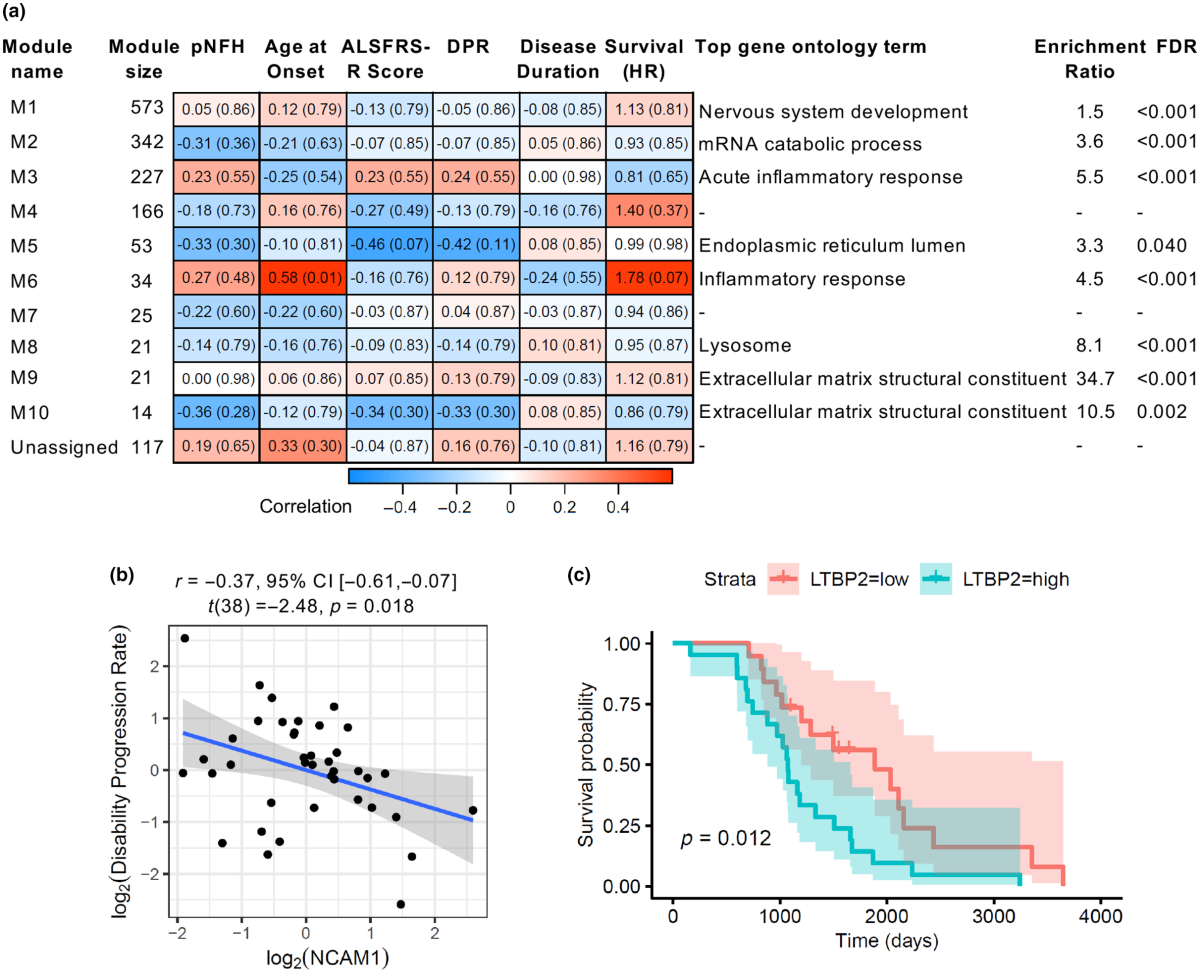
Network analysis identifies inflammatory and endoplasmic reticulum protein modules as associated with disability progression rate and survival respectively. (a) Table showing module allocation in Weighted Correlation Network Analysis, alongside correlation with phosphorylated neurofilament heavy (pNFH) in CSF from ELISA data, age at onset, ALS (ALSFRS‐R), disability progression rate (DPR), disease duration and univariate hazard ratios. Most significantly enriched gene ontology terms are also shown for each module, with Enrichment ratio and adjusted p‐value (FDR, Benjamini‐Hochberg correction). (b) Correlation of neural cell adhesion molecule 1 (NCAM1) (the most highly connected intramodular hub proteins for module M5) with disability progression rate. (c) Kaplan–Meier survival analysis for latent transforming growth factor beta binding protein (LTBP2) (the most highly connected intramodular hub protein for module M6), stratified by mean protein abundance. *p*‐value for log‐rank test. ALSFRS‐R, revised ALS functional rating scale; CI, confidence interval; DPR, disability progression rate; FDR, false discovery rate; HR, hazard ratio; LTBP2, latent transforming growth factor binding protein 2; N/A, not assigned; NCAM1, neural cell adhesion molecule 1; pNFH, phosphorylated neurofilament heavy chain.

We next undertook weighted correlation network analysis as a systems‐based analysis of correlated changes in protein abundance, to identify whether there were changes in biological pathways represented in the proteomic data (Langfelder & Horvath, 2008). This analysis classified 1476 of 1593 protein groups into 10 modules, varying in size from 14 to 573 protein groups, with 117 unassigned (Figure S2a; Figure 5a). Gene ontology overrepresentation analysis was used to identify the main biological mechanisms reflected by individual modules (Figure 5a, full data shown in Data S1). Of the identified differentially abundant proteins, CHIT1, CHI3L2, FCGR3A, CFD and VDAC1 were unassigned. CHI3L1 was contained within module M6 (34 proteins) that was enriched for inflammatory response (enrichment ratio = 4.5, FDR‐adjusted *p* < 0.001), whilst both UCHL1 and SERPINA3 were within the largest (573 proteins) module (M1) that was enriched for nervous system development (enrichment ratio = 1.5, FDR‐adjusted *p* < 0.001). Of other proteins of interest, almost all apolipoproteins (APOB, APOA1/A2/A4 and APOC1/C2/C3) were found within module M3 (227 proteins), enriched in acute inflammatory response (enrichment ratio = 5.5, FDR‐adjusted *p* < 0.001), demonstrating the capability of network approaches to identify biologically relevant subnetworks represented in the CSF.

Differences in module protein expression were assessed by comparison of module eigenproteins, but no differences between samples from people with ALS and healthy controls or disease controls were seen, likely because of high variability (Figure S2c). Two modules (M5 and M6) showed strong associations with disability progression rate or survival (Figure 5a). Module M5 (53 proteins) was enriched for endoplasmic reticulum lumen proteins (enrichment ratio = 3.3, FDR‐adjusted *p* = 0.040), and correlated with disability progression rate (*r* = −0.42, FDR‐adjusted *p* = 0.109). Module M6 (inflammatory response) was associated with survival (hazard ratio = 1.78, FDR‐adjusted *p* = 0.065) and with age at onset (*r* = 0.58, FDR‐adjusted *p* = 0.005). Log‐rank analysis of Kaplan–Meier survival of ALS patients, based on module eigenprotein expression dichotomised by mean level in the inflammatory response module confirmed a significant association with survival (M6: log‐rank *p* = 0.019, Figure S2d).

**FIGURE 6:**
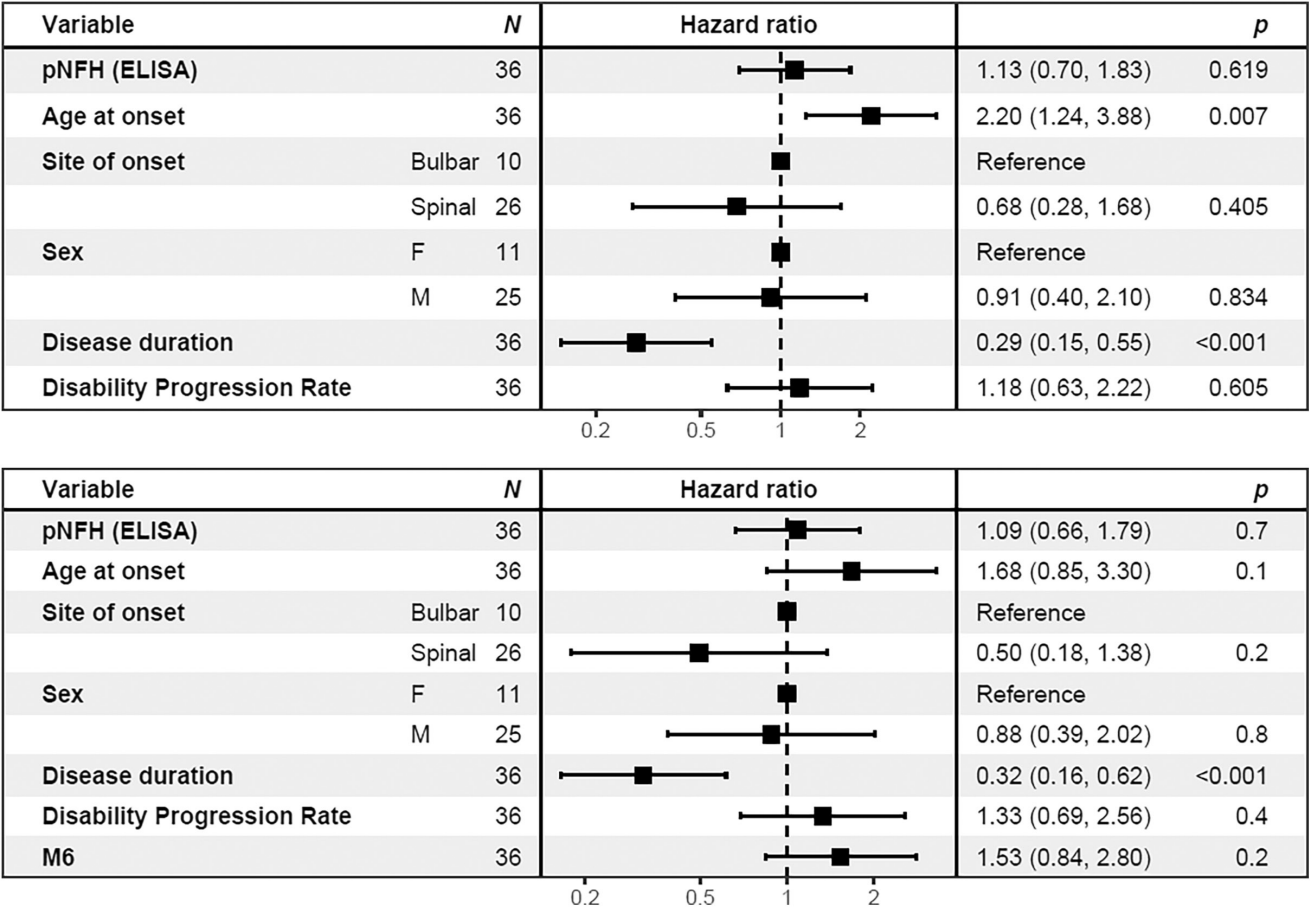
Inflammatory protein module M6 does not show an independent effect on survival. ALS patients at the first visit, with or without the inclusion of inflammatory protein module eigenvalues as a variable. CI, confidence interval; HR, Hazard ratiol; pNFH, phosphorylated neurofilament heavy. Hazard ratios represent a rise per one standard deviation.

To further validate the network analysis approach, we selected the most highly interconnected intramodular hub protein for each primary module of interest; Neural Cell Adhesion Molecule 1 (NCAM1) for the endoplasmic reticulum module, and latent transforming growth factor beta binding protein 2 (LTBP2) for the inflammatory response module; and confirmed that these single proteins also showed a strong association with disability progression (NCAM1) or survival (LTBP2; Figure 5b,c). Further Cox proportional hazards models were constructed incorporating available first‐visit clinical characteristics in addition to M5 and M6 module eigenvalues, or abundance of module hub proteins (NCAM1, LTBP2), with or without inclusion of pNFH. Assessment of model fit with Akaike's Information Criterion (AIC) showed the best model contained only pNFH in addition to clinical characteristics, followed by the model containing the inflammatory model (M6) eigenvalue. In this next best model, the age of onset became insignificant as an independent variable, further demonstrating the strong interaction between inflammatory involvement and onset age as risk variables (Figure 6). These data demonstrated the involvement of different biological processes, such as endoplasmic reticulum stress and inflammatory response in disease prognosis.

## 4 DISCUSSION

Using a simplified pre‐analytical preparation protocol combined with label‐free LC–MS/MS in DIA mode, we were able to quantify a total of 1832 protein groups from only 50 μL of CSF and identified alterations in the levels of individual CSF proteins and network‐level changes in people with ALS. This depth is similar to that achieved with DIA in recent studies for non‐fractionated CSF (1493–1732 proteins, Cho et al., 2021; Karayel et al., 2022), although somewhat lower than previous studies in ALS that have made use of labelling methods (1936–2303 proteins, Barschke et al., 2020; Oeckl et al., 2020; Oh et al., 2023). Importantly, however, the DIA approach resulted in a much lower rate of missing values than typically achieved with DDA, with between‐group comparisons carried out on 1593 proteins after a stringent (50% per group) filtering threshold, thus allowing for highly robust comparisons. The label‐free approach also lowers hands‐on processing requirements, reducing the potential for the introduction of contamination, technical variability, and requirement for batch normalisation, facilitating higher throughput sample preparation.

This simplified preparation method replicated the identification of differentially abundant proteins seen in these other studies in ALS, notably CHIT1, CHI3L1, UCHL1 and SERPINA3 (Oeckl et al., 2020; Oh et al., 2023; Thompson et al., 2018). One particular exception in our study was the lack of identification of differential abundance of neurofilament proteins (NEFH, NEFM, NEFL). In the full dataset, NEFH was identified in 17 samples in total (all ALS), but removed because of the strict 50% per group filtering threshold, whilst NEFM and NEFL were not detected. Their absence may be reflective of the low absolute abundance of neurofilament proteins in CSF, poor digestion with the preparation method employed, or poor ionisation/high fragmentation in mass spectrometry.

Whilst the mammalian chitinase proteins (CHIT1, CHI3L1 and CHI3L2) have been identified as increased in abundance in CSF of ALS patients, mixed data exists on their prognostic value. This methodology demonstrated an association of CHIT1 with disability progression rate, but not survival, in agreement with a previous study (Steinacker et al., 2018). A further multicentre study found CHIT1 to be an independent predictor of survival in late (>6 months), but not early (<6 months) symptomatic ALS (Steinacker et al., 2021). However, CHIT1 levels in late symptomatic, but not early symptomatic disease were found to be significantly lower in individuals with a 24‐bp duplication polymorphism of CHIT1, which is known to reduce levels of CHIT1 in CSF, and is common within European populations (Malaguarnera et al., 2003; Steinacker et al., 2021). In our small study the prevalence of this polymorphism within people with ALS was unknown, so may underlie the lack of association with survival observed.

Unlike CHIT1, CHI3L1 and CHI3L2 were not significantly increased when comparing ALS to disease controls and showed no link with either the rate of disability progression or survival. In agreement with this, an independent study also found only CHIT1 to be raised in ALS CSF when compared to disease (rather than healthy) controls (Vu et al., 2020). CHI3L1 has also been shown to be increased in CSF in multiple other neurological diseases including multiple sclerosis and Alzheimer's disease (Craig‐Schapiro et al., 2010; Hinsinger et al., 2015; Vu et al., 2020).

In our network analysis, one module (M6) was highly enriched for ‘inflammatory response’ and showed a significant survival association in Kaplan–Meier analysis but not a significant independent effect in proportional hazards models. M6 was also associated with the age of symptom onset. This inflammatory module contained multiple markers of both classically reactive microglia (Complement C1QA/B/C, CD14) and reactive astrocytes (HSPB1, THBS1, LTBP2; Escartin et al., 2021; Liddelow et al., 2017; Ziff et al., 2022). In agreement with these findings, clustering of ALS patients based on post‐mortem brain transcriptomic data indicated similar associations between inflammatory signatures and age of disease onset (Eshima et al., 2023). The strong increase in abundance of chitinases in CSF has been assumed to be indicative of inflammatory involvement, but in our analysis, only CHI3L1 was contained within this inflammatory protein module (Brettschneider et al., 2012; Varghese et al., 2013). This is in agreement with other studies showing that increased CHI3L1 expression represents an age‐related inflammatory response that is common in many neurodegenerative conditions (Karayel et al., 2022; Sanfilippo et al., 2019).

Unlike CHI3L1, CHIT1 and CHI3L2 were not classified in network analysis, which may suggest their secretion is less strongly correlated with broader inflammatory processes. Whilst CHIT1 colocalises with IBA1 and CD68 in the spinal cord, indicating macrophage or microglial origin, the activation state of these cells is not defined (Steinacker et al., 2018). Indeed, the induction of inflammatory response by lipopolysaccharide treatment has been found to induce a strong increase in expression of CHI3L1, but not CHIT1, in macrophages, whilst CHIT1 expression was strongly increased by interleukin‐4 which is considered as an anti‐inflammatory cytokine (Di Rosa et al., 2013). Furthermore, in the lysosomal storage disorder Gaucher disease, for which secreted CHIT1 is a key diagnostic marker (Hollak et al., 1994), the characteristically lipid‐laden macrophages show increases in the abundance of chemokine CCL18 and Glycoprotein nonmetastatic melanoma protein B (GPNMB) that are typically more indicative of alternatively activated microglia (Hüttenrauch et al., 2018; Kramer et al., 2016; Zigdon et al., 2015). Whilst significantly increased abundance was not found in this study, GPNMB has also been identified as increased in CSF of ALS patients in other studies (Oeckl et al., 2020; Oh et al., 2023). Given that in transgenic *SOD1* models of ALS there is evidence for a shift from alternatively activated to classically activated microglia over the disease course (Gravel et al., 2016; Liao et al., 2012), defining the specific activation state of the cellular source of all the chitinase proteins in CSF will further contribute to understanding of the contribution of neuroinflammation to the clinical heterogeneity of the disease.

Network analysis also revealed an association of a set of endoplasmic reticulum enriched proteins with disability progression rate, in particular: Endoplasmic reticulum chaperone BiP (HSPA5, BiP or GRP78), calreticulin (CALR), and protein disulfide isomerase 4 (PDIA4), which are all associated with induction of endoplasmic reticulum stress. Endoplasmic reticulum stress is thought to be an important early factor in cellular dysfunction in both sporadic and *C9orf72* ALS, with a direct interaction between TDP‐43 and BiP also demonstrated (Dafinca et al., 2016, 2021; Feneberg et al., 2020; François‐Moutal et al., 2022; Pilotto et al., 2022; Sasaki, 2010). Our study therefore provides additional novel data indicating a signature of this endoplasmic reticulum dysfunction is evident in CSF, which may be of importance in understanding prognostic heterogeneity in ALS.

UCHL1 showed both a highly significant increase in abundance and significant associations with disability progression rate and survival. UCHL1 was previously identified as increased in abundance in CSF of ALS patients in a targeted multiple reaction monitoring mass spectrometry, non‐targeted mass spectrometry, Luminex and Simoa assays (Li et al., 2020; Oeckl et al., 2020; Zhu et al., 2019). Furthermore, UCHL1 levels in serum were found to be tightly linked to CSF abundance in a small study of 23 ALS patients (Li et al., 2020). UCHL1 is very highly expressed in the brain, including in motor neurons and is estimated to constitute 1%–5% of total neuronal protein, where it plays an essential role in regulating the levels of free ubiquitin required for the functioning of the ubiquitin‐proteasome system (Bishop et al., 2016). UCHL1 mutation or knockout mice exhibit axonal degeneration, resulting in ataxic phenotypes, demonstrating its importance in maintaining neuronal integrity (Bilguvar et al., 2013; Chen et al., 2010; Walters et al., 2008). UCHL1 is also expressed in microglia and has been suggested to be a regulator of inflammasome activation (Liang et al., 2023). Loss‐of‐function mutations and oxidative modifications of UCHL1 have also been linked to Parkinson's disease and Alzheimer's disease (Butterfield et al., 2006; Liu et al., 2015; Ragland et al., 2009). UCHL1 also exists in both cytosolic and membrane‐associated forms, the membrane form being particularly enriched in neurons (Bishop et al., 2014, 2016). In a study of the membrane fraction of post‐mortem frontal cortex tissue, UCHL1 was found to be increased in abundance in Alzheimer's disease, indicating disease relevance of cytosolic versus membrane‐association (Donovan et al., 2012). The exact nature of the associated membrane is unclear but has been suggested to be endoplasmic reticulum, with further evidence suggesting a role in regulating mitochondrial morphology, through an association with outer mitochondrial membrane protein VDAC1, processes which have known relevance in the pathogenesis of ALS (Dafinca et al., 2021; Gao et al., 2020; Liu et al., 2009). Therefore, distinguishing the relative abundance of these membrane and cytosolic forms of UCHL1 in ALS may hold great value in defining its diagnostic and prognostic value.

In conclusion, our study demonstrated the performance of label‐free LC–MS/MS in DIA mode in high‐depth proteomic analysis of CSF samples for biomarker discovery. It identified endoplasmic reticulum and inflammatory protein signatures which hold the potential to help determine the biochemical basis of the clinical heterogeneity in disease onset, progression and survival in ALS.

## AUTHOR CONTRIBUTIONS

**Elizabeth Dellar:** Conceptualization; data curation; formal analysis; investigation; methodology; visualization; writing – original draft; writing – review and editing. **Iolanda Vendrell:** Conceptualization; data curation; formal analysis; investigation; methodology; writing – review and editing. **Kevin Talbot:** Conceptualization; resources; supervision; writing – review and editing. **Benedikt M. Kessler:** Conceptualization; methodology; resources; writing – review and editing. **Roman Fischer:** Conceptualization; formal analysis; investigation; methodology; resources; supervision; writing – review and editing. **Martin R. Turner:** Conceptualization; data curation; investigation; methodology; resources; supervision; writing – review and editing. **Alexander G. Thompson:** Conceptualization; formal analysis; investigation; methodology; resources; supervision; writing – review and editing.

## CONFLICT OF INTEREST STATEMENT

The authors have no conflict of interest to declare.

### PEER REVIEW

The peer review history for this article is available at https://www.webofscience.com/api/gateway/wos/peer‐review/10.1111/jnc.16030.

## Supporting information


Figure S1.
Figure S2.
Table S1.
Table S2.

Data S1.

## Data Availability

The mass spectrometry proteomics data have been deposited to the ProteomeXchange Consortium via the PRIDE (Perez‐Riverol et al., 2019) partner repository (https://proteomecentral.proteomexchange.org/cgi/GetDataset) with the dataset identifier PXD044274.
