# Supplementary material for: Data‐independent acquisition proteomics of cerebrospinal fluid implicates endoplasmic reticulum and inflammatory mechanisms in amyotrophic lateral sclerosis
Source: J Neurochem. 2023 Dec 12;168(2):115–27. doi: 10.1111/jnc.16030 (PMC10952667; doi:10.1111/jnc.16030)
Supplement: Supplementary file 1 — Figure S1. Figure S2. Table S1. Table S2. [file JNC-168-115-s002.pdf]

Data-independent acquisition proteomics of cerebrospinal fluid implicates endoplasmic reticulum and inflammatory mechanisms in amyotrophic lateral sclerosis

Elizabeth R Dellar [1], Iolanda Vendrell [2,4], Kevin Talbot [1.3], Benedikt M Kessler [2,4], Roman Fischer [2,4], Martin R Turner [1], Alexander G Thompson [#1].

Affiliations:

[1] Nuffield Department of Clinical Neurosciences, University of Oxford, UK
[2] Target Discovery Institute, Centre for Medicines Discovery, Nuffield Department of Medicine, University of Oxford, UK
[3] Kavli Institute for Nanoscience Discovery, University of Oxford, UK
[4] Chinese Academy of Medical Sciences Oxford Institute, Nuffield Department of Medicine, University of Oxford, UK

Disease controls (n=8)
Conduction block neuropathy (n=3)
Multifocal acquired demyelinating sensory and motor neuropathy (n=1)
Progressive multiple sclerosis (n=1)
Cervical myelopathy (n=1)
Non-progressive unilateral upper limb weakness (n=1)
Gait initiation failure (n=1)

Supplementary Table 1: Diagnoses for disease controls

m/z	z	Isolation Window (m/z)	Normalized AGC Target (%)
361.5	2	23	10
381.5	2	19	10
398.5	2	17	10
413.5	2	15	10
427	2	14	10
440	2	14	10
452.5	2	13	10
464	2	12	10
475.5	2	13	10
487.5	2	13	10
499.5	2	13	10
511	2	12	10
522.5	2	13	10
534.5	2	13	10
546.5	2	13	10
558	2	12	10
569.5	2	13	10
581.5	2	13	10
594.5	2	15	10
607.5	2	13	10
619.5	2	13	10
633	2	16	10
647.5	2	15	10
661.5	2	15	10
675.5	2	15	10
690	2	16	10
705.5	2	17	10
722.5	2	19	10
741	2	20	10
760.5	2	21	10
781	2	22	10
803	2	24	10
826.5	2	25	10
853	2	30	10
883	2	32	10
918.5	2	41	10
960.5	2	45	10
1013	2	62	10
1085	2	84	10
1388	2	524	10

Supplementary Table 2: DIA scan window widths

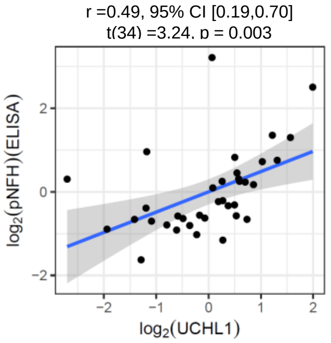

Supplementary Figure 1: Correlation between LC-MS/MS UCHL1 and ELISA pNFH

pNFH = phosphorylated neurofilament heavy, Ubiquitin carboxyl-terminal hydrolase isozyme L1.

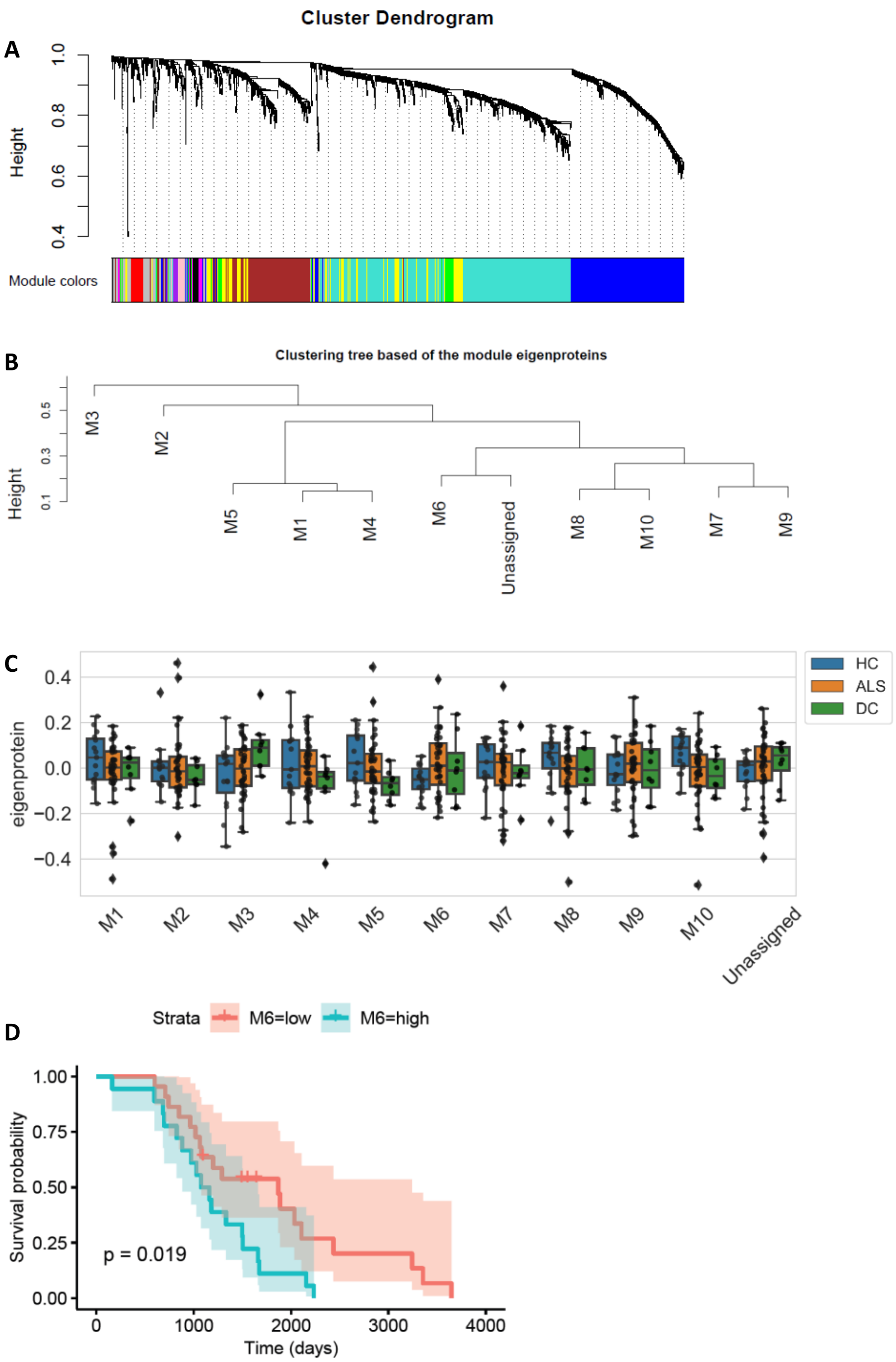

Supplementary Figure 2: No differences in module eigenvalues in Weighted Correlation Network Analysis in ALS versus controls.

(A) Cluster dendrogram indicating module allocation of proteins. **(B)** Clustering tree showing degree of dissimilarity between protein modules. **(C)** Relative expression of module eigenprotein in each group. HC = healthy control, DC = disease control. No differences with ALS group statistically significant. **(D)** Kaplan-Meier survival analysis for M6 eigenprotein values, stratified by mean value. p-value for log-rank test.
